# Supplementary material for: Synthetic mycobacterial molecular patterns partially complete Freund’s adjuvant
Source: Sci Rep. 2020 Apr 3;10:5874. doi: 10.1038/s41598-020-62543-5 (PMC7125112; doi:10.1038/s41598-020-62543-5)
Supplement: Supplementary file 1 — Supplementary information. [file 41598_2020_62543_MOESM1_ESM.pdf]

Supplemental Information

Title

Synthetic mycobacterial molecular patterns partially complete Freund's adjuvant

Authors/Affiliations

Jean-Yves Dubé [1,2,3*], Fiona McIntosh [2,3], Juan G. Zarruk [4], Samuel David [4], Jérôme Nigou [5], Marcel A. Behr [1,2,3,6*]

1, Department of Microbiology and Immunology, McGill University. 2, Infectious diseases and Immunity in Global Health Program, Research Institute of the McGill University Health Centre. 3, McGill International TB Centre. 4, Centre for Research in Neuroscience, Research Institute of the McGill University Health Centre. 5, Institut de Pharmacologie et de Biologie Structurale, Université de Toulouse, CNRS, Université Paul Sabatier, Toulouse, France. 6, Department of Medicine, McGill University Health Centre, Montréal, Canada.

Contact Info*
jean-yves.dube@mail.mcgill.ca
marcel.behr@mcgill.ca (Lead Contact)
McGill University Health Centre
1001 boul. Décarie
Glen Site Block E, Office #EM3.3212
Montréal, Québec, Canada
H4A 3J1

Supplemental Figure Titles and Legends

Figure S1. Flow cytometry gating strategies for lymph node cells. A, gating to quantify cytokine-producing CD4+CD8- T cells. Shown are representative gating and data from an OVA-stimulated sample (CFA-immunized WT mouse). For comparison, cytokine production for the corresponding unstimulated sample is included. **B**, gating to identify lymph node cell subsets. Shown is gating on a representative sample (CFA-immunized WT mouse). pDCs are B220+Ly6C+MHC-II+CD11b-CD11c+ cells. B cells are B220+Ly6C-CD11b-CD11c-CD4-CD8a- cells. cDCs are B220-MHC-II^{hi}Ly6C-CD11c+ cells (analyzed subsets are CD11b+/-). CD4+ T cells are B220-CD11b-CD4+CD8a- cells. CD8+ T cells are B220-CD11b-CD4-CD8a+ cells. Monocytes are B220-CD11b+Ly6C+SSC^{lo} cells. PMNs are B220-CD11b+Ly6C^{med}SSC^{hi} CD11c- cells.

Figure S2. Addition data on CFA-dependent cell-mediated immune responses as a function of mycobacterial *namH*. These data are from the same experiments shown in fig. 1C (refer to fig. 1C legend for details). **A-B**, Total numbers (from two lymph nodes) of cytokine-producing CD4+CD8- lymph node cells of mice immunized against OVA with H37Rv, H37Rv Δ*namH*, or IFA alone. Shown are averages +/- SEM. p-values were calculated with two-tailed student's *t*-tests. **p<0.01. **C-D**, Contribution of *namH* to the mycobacterial portion of OVA-specific IFN-γ elicited by CFA. **C**, result was obtained from %IFN-γ+ data by subtracting the average IFA background from all CFA data, and plotting the results as % of IFA+H37Rv 'wild-type'. **D**, result was obtained from # IFN-γ+ data by subtracting the average IFA background from all CFA data, and plotting the results as % of IFA+H37Rv 'wild-type'. Shown are averages +/- SEM. p-values were calculated with two-tailed student's t-tests. *p<0.05. **E**, results separated by experimental run for the data presented in fig. 1C and fig. S2A-B. Shown are averages +/- SEM.

Figure S3. CFA-dependent IFN-γ response by ELISpot as a function of host *Nod2*. A-B, IFN-γ ELISpot of inguinal lymph node cells mice immunized against OVA seven days prior, produced in an independent experiment. **A**, number of IFN-γ spot-forming cells per one million cells. **B**, total number of IFN-γ spot-forming cells per two inguinal lymph nodes. Shown are averages +/- SEM. p-values were calculated with two-tailed student's *t*-tests. *p<0.05. For CFA *Nod2*+/+, CFA *Nod2*-/-, IFA *Nod2*+/+ and IFA *Nod2*-/-, N = 6, 8, 6 and 6 mice, respectively. **C**, flow cytometry of inguinal lymph node cells mice immunized against OVA seven days prior, produced in an independent experiment similar to that in fig. 2A-B. **D**, results separated by experimental run for the data presented in fig. 2C-D and fig. S4C-D. Shown are averages +/- SEM.

Figure S4. Additional data on CFA-dependent cell-mediated immune responses as a function of host *Nod2* and Mincle. These data are from the same experiments shown in fig. 2 (refer to fig. 2 legend for details). **A-F**, Total numbers (from two lymph nodes) of cytokine-producing CD4+CD8- lymph node cells of mice immunized against OVA with the indicated adjuvant as a function of: **A-B**, *Nod2*; **C-D**, *Mincle*; **E-F**, *Mincle* and *Nod2* together. Shown are averages +/- SEM. p-values were calculated with two-tailed student's *t*-tests. *p<0.05; ns, not significant, p>0.05.

Figure S5. Additional data on MHC-II expression and costimulatory molecule upregulation by BMDCs stimulated with GlcC14C18 and MDPs. **A**, percentage of cells expressing MHC-II at high levels (according to gate in fig. 3B) after 48 hours of stimulation with the indicated MAMPs. **B-E**, median fluorescence intensity of **B**, MHC-II; **C**, CD40; **D**, CD80; **E**, CD86 on CD11b+CD11c+MHC-II^{hi} cells after 48 hours of stimulation with the indicated MAMPs (use legend of panel A). Shown are averages +/- SD of 3 individually stimulated and assayed cultures. **F-I**, histograms of CD11b+CD11c+MHC-II^{hi} cells demonstrating expression levels of **F**, MHC-II; **G**, CD40; **H**, CD80; **I**, CD86, for both timepoints (FMOC is not shown for MHC-II because the analysis required MHC-II gating).

Figure S6. Synthetic adjuvant-dependent IFN-γ responses compared to CFA by ELISpot. **A-B**, IFN-γ ELISpot of inguinal lymph node cells of mice immunized against OVA with the indicated adjuvant seven days prior. **A**, number of IFN-γ spot-forming cells per one million cells. **B**, total number of IFN-γ spot-forming cells per two inguinal lymph nodes. Data was pooled from two independent experiments. Shown are averages +/- SEM, where for IFA, IFA + 3 µg *N*-glycolyl MDP, IFA + 10 µg *N*-glycolyl MDP, IFA + 30 µg *N*-glycolyl MDP, IFA + 100 µg *N*-glycolyl MDP and CFA, N = 11, 6, 12, 11, 6 and 11 mice, respectively. **C-D**, These data are from the same mice used in fig. 4B (refer to fig. 4B legend for details). **C**, number of IFN-γ spot-forming cells per one million cells. **D**, total number of IFN-γ spot-forming cells per two inguinal lymph nodes. Shown are averages +/- SEM. In comparing IFA + 30 µg MDP to IFA + MDP + 10 or 30 µg GlcC14C18, p-values were calculated using Dunnett's T3 multiple comparisons test. *$p<0.05$; **$p<0.01$; ns, not significant, $p>0.05$.

Figure S7. Lymph node cell subset numbers after immunization with synthetic adjuvants. These data are from the same mice used in fig. 5 (refer to fig. 5 legend for details of the experiment). **A-F,** From two inguinal lymph nodes at 4 or 7 days post-immunization, shown are total numbers of extracted **A**, B cells; **B**, CD4+ T cells; **C**, CD8+ T cells; **D**, plasmacytoid dendritic cells (pDCs); **E**, monocytes; **F**, polymorphonuclear cells (PMNs). Data are graphed as averages +/- SEM.

Figure S8. Additional statistics for RR-EAE. These data are from the same set shown in fig. 5 (refer to fig. 5 legend for details). **A**, average weight of mice over time +/- SEM. **B**, maximum EAE score reached on any day by mice, as of day 28. **C**, disease course of mice selected for spinal cord histopathology. **D-G**, Statistics for RR-EAE induced by IFA+TDM+MDP. **D**, average EAE score +/- SEM over time of mice induced with CFA (N=13) or IFA + 1 µg TDM + 30 µg *N*-glycolyl MDP (N=13). Mice were euthanized on day 27 post injection. **E**, cumulative EAE score, obtained by adding the EAE score of each mouse over each of the 27 days. Lines represent averages +/- SEM. **F**, average weight of mice over time +/- SEM. **G**, maximum EAE score reached on any day by mice, as of day 27.

Supplemental Tables

Table S1. Flow cytometry antibodies for lymph node cell cytokines

Target	Supplier	Clone	Fluorochrome*
CD3ε	BD	145-2C11	PE
CD4	BD	GK1.5	BV786
CD8α	BD	53-6.7	BV711
CD19	Biolegend	6D5	PE-Dazzle594
B220/CD45R	BD	RA3-6B2	BUV737
IFN-γ	Biolegend	XMG1.2	APC
IL-2	BD	JES6-5H4	BV605
IL-4	eBiosciences	BVD6-24G2	PE-Cy7
IL-17A	BD	TC11-18H10	BUV395
IL-10	BD	JES5-16E3	FITC

*Viability dye: LIVE/DEAD™ Fixable Violet Dead Cell Stain (ThermoFisher Scientific)

Table S2. Flow cytometry antibodies for lymph node cell DC and subset analysis

Target	Supplier	Clone	Fluorochrome*
B220/CD45R	BD	RA3-6B2	BUV737
CD4	BD	GK1.5	BV786
CD8α	BD	53-6.7	BV711
CD11b	Biolegend	M1/70	BV605
CD11c	BD	HL3	FITC
CD40	Biolegend	3/23	PE-Dazzle594
CD80	Biolegend	16-10A1	PE
CD86	Biolegend	GL1	PE-Cy7
CD209	BD	5H10	BUV395
F4/80	Biolegend	BM8	APC
Ly6C	Biolegend	HK1.4	APC-Cy7
MHC-I (H-2K^b)	Biolegend	AF6-88.5	BV510
MHC-II (I-A^b)	Biolegend	AF6-120.1	PerCP-Cy5.5

*Viability dye: LIVE/DEAD™ Fixable Violet Dead Cell Stain (ThermoFisher Scientific)

Table S3. Flow cytometry antibodies for BMDCs

Target	Supplier	Clone	Fluorochrome*
CD11b	Biolegend	M1/70	PerCP-Cy5.5
CD11c	BD	HL3	FITC
CD40	Biolegend	3/23	PE-Dazzle594
CD80	Biolegend	16-10A1	PE
CD86	Biolegend	GL1	PE-Cy7
MHC-II (I-A^b)	Biolegend	AF6-120.1	APC

*Viability dye: LIVE/DEAD™ Fixable Violet Dead Cell Stain (ThermoFisher Scientific)

FIG. S1

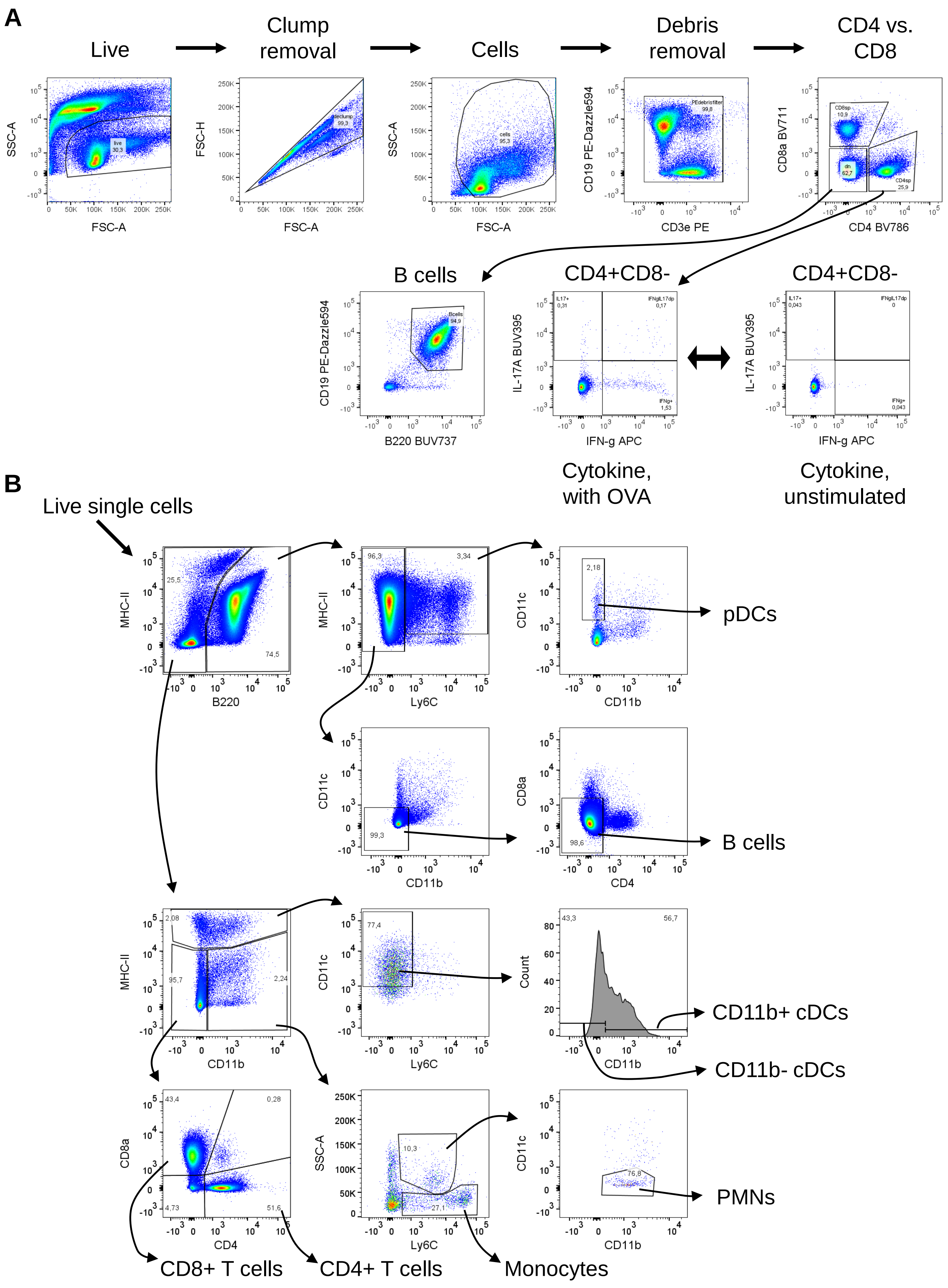
A
Live
Clump removal
Cells
Debris removal
CD4 vs. CD8
SSC-A
FSC-A
FSC-H
CD19 PE-Dazzle594
CD3e PE
CD8a BV711
CD4 BV786
B cells
CD4+CD8-
B220 BUV737
IL-17A BUV395
IFN-g APC
Cytokine, with OVA
Cytokine, unstimulated
B
Live single cells
MHC-II
B220
Ly6C
CD11c
CD11b
pDCs
CD8a
CD4
B cells
Count
CD11b+ cDCs
CD11b- cDCs
PMNs
CD8+ T cells
CD4+ T cells
Monocytes

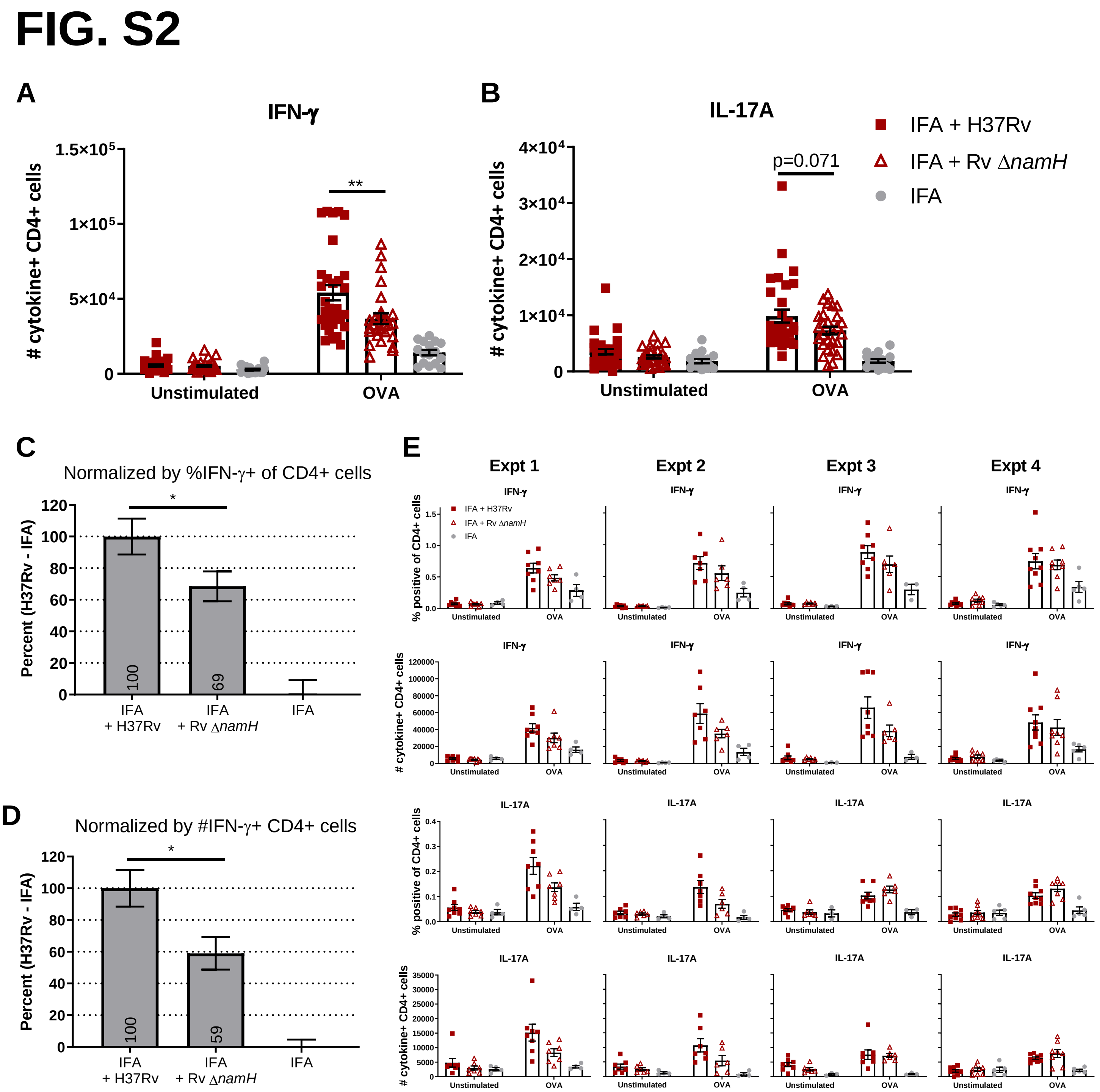
FIG. S2
A
IFN-γ
cytokine+ CD4+ cells
**
Unstimulated
OVA
B
IL-17A
p=0.071
IFA + H37Rv
IFA + Rv ΔnamH
IFA
C
Normalized by %IFN-γ+ of CD4+ cells
Percent (H37Rv - IFA)
*
100
69
IFA + H37Rv
IFA + Rv ΔnamH
IFA
D
Normalized by #IFN-γ+ CD4+ cells
Percent (H37Rv - IFA)
*
100
59
IFA + H37Rv
IFA + Rv ΔnamH
IFA
E
Expt 1
Expt 2
Expt 3
Expt 4
IFN-γ
IL-17A
% positive of CD4+ cells
# cytokine+ CD4+ cells
Unstimulated
OVA

FIG. S3

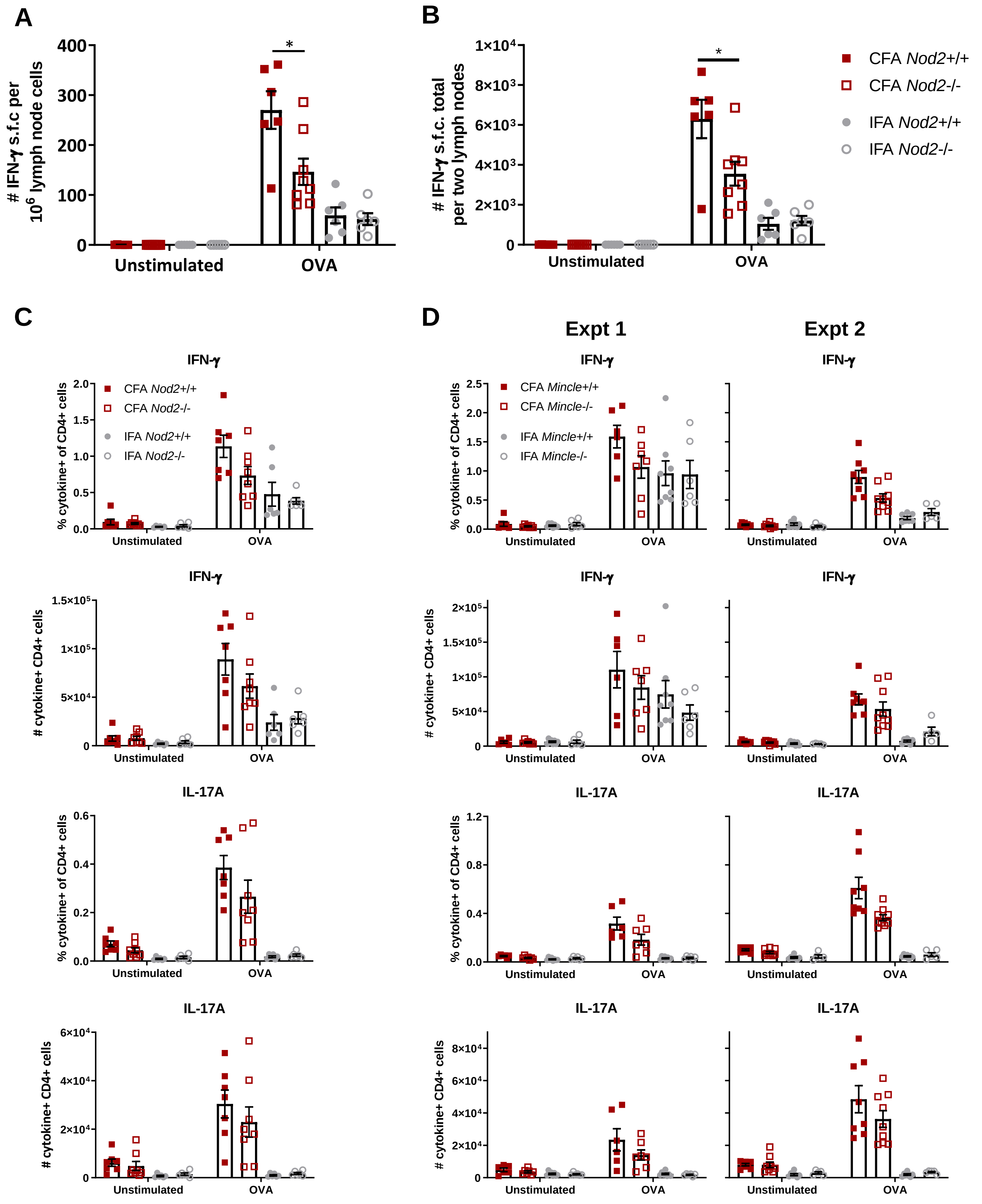
A
IFN-γ s.f.c per 10^6 lymph node cells
400
300
200
100
0
Unstimulated
OVA
*
B
IFN-γ s.f.c. total per two lymph nodes
1×10^4
8×10^3
6×10^3
4×10^3
2×10^3
0
Unstimulated
OVA
*
CFA Nod2+/+
CFA Nod2-/-
IFA Nod2+/+
IFA Nod2-/-
C
IFN-γ
% cytokine+ of CD4+ cells
2.0
1.5
1.0
0.5
0.0
CFA Nod2+/+
CFA Nod2-/-
IFA Nod2+/+
IFA Nod2-/-
cytokine+ CD4+ cells
1.5×10^5
1×10^5
5×10^4
IL-17A
0.6
0.4
0.2
6×10^4
4×10^4
2×10^4
D
Expt 1
Expt 2
IFN-γ
2.5
CFA Mincle+/+
CFA Mincle-/-
IFA Mincle+/+
IFA Mincle-/-
2×10^5
1.5×10^5
1×10^5
5×10^4
IL-17A
1.2
0.8
0.4
8×10^4
6×10^4
4×10^4
2×10^4
Unstimulated
OVA

FIG. S4

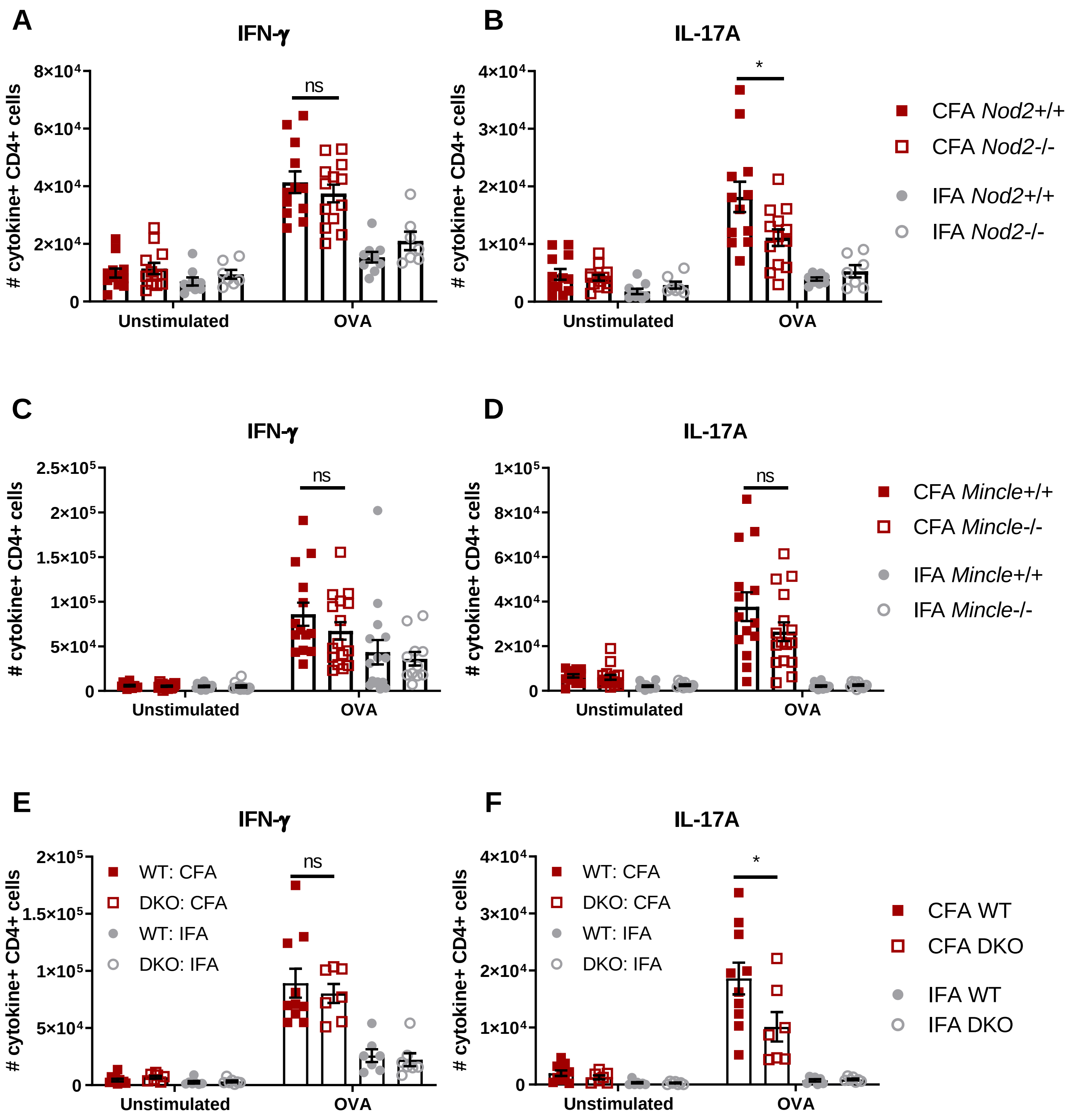
A
IFN-γ
cytokine+ CD4+ cells
8×10⁴
6×10⁴
4×10⁴
2×10⁴
0
ns
Unstimulated
OVA
B
IL-17A
cytokine+ CD4+ cells
4×10⁴
3×10⁴
2×10⁴
1×10⁴
0
*
Unstimulated
OVA
CFA Nod2+/+
CFA Nod2-/-
IFA Nod2+/+
IFA Nod2-/-
C
IFN-γ
cytokine+ CD4+ cells
2.5×10⁵
2×10⁵
1.5×10⁵
1×10⁵
5×10⁴
0
ns
Unstimulated
OVA
D
IL-17A
cytokine+ CD4+ cells
1×10⁵
8×10⁴
6×10⁴
4×10⁴
2×10⁴
0
ns
Unstimulated
OVA
CFA Mincle+/+
CFA Mincle-/-
IFA Mincle+/+
IFA Mincle-/-
E
IFN-γ
cytokine+ CD4+ cells
2×10⁵
1.5×10⁵
1×10⁵
5×10⁴
0
WT: CFA
DKO: CFA
WT: IFA
DKO: IFA
ns
Unstimulated
OVA
F
IL-17A
cytokine+ CD4+ cells
4×10⁴
3×10⁴
2×10⁴
1×10⁴
0
WT: CFA
DKO: CFA
WT: IFA
DKO: IFA
*
Unstimulated
OVA
CFA WT
CFA DKO
IFA WT
IFA DKO

FIG. S5

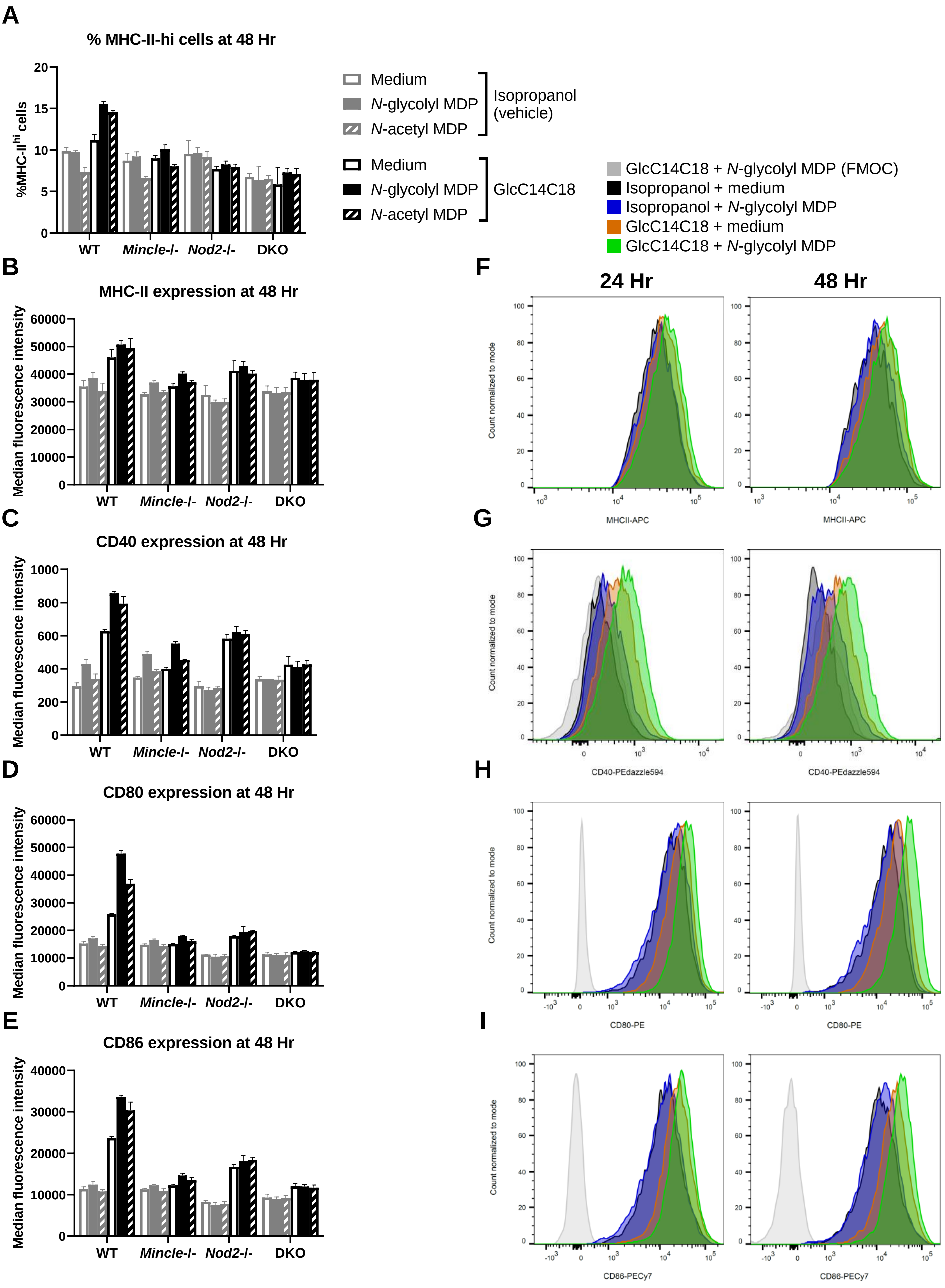

A
% MHC-II-hi cells at 48 Hr
%MHC-II^{hi} cells
WT
Mincle-/-
Nod2-/-
DKO
Medium
N-glycolyl MDP
N-acetyl MDP
Isopropanol (vehicle)
Medium
N-glycolyl MDP
N-acetyl MDP
GlcC14C18
GlcC14C18 + N-glycolyl MDP (FMOC)
Isopropanol + medium
Isopropanol + N-glycolyl MDP
GlcC14C18 + medium
GlcC14C18 + N-glycolyl MDP
B
MHC-II expression at 48 Hr
Median fluorescence intensity
C
CD40 expression at 48 Hr
D
CD80 expression at 48 Hr
E
CD86 expression at 48 Hr
F
24 Hr
48 Hr
Count normalized to mode
MHCII-APC
G
CD40-PEdazzle594
H
CD80-PE
I
CD86-PECy7

FIG. S6

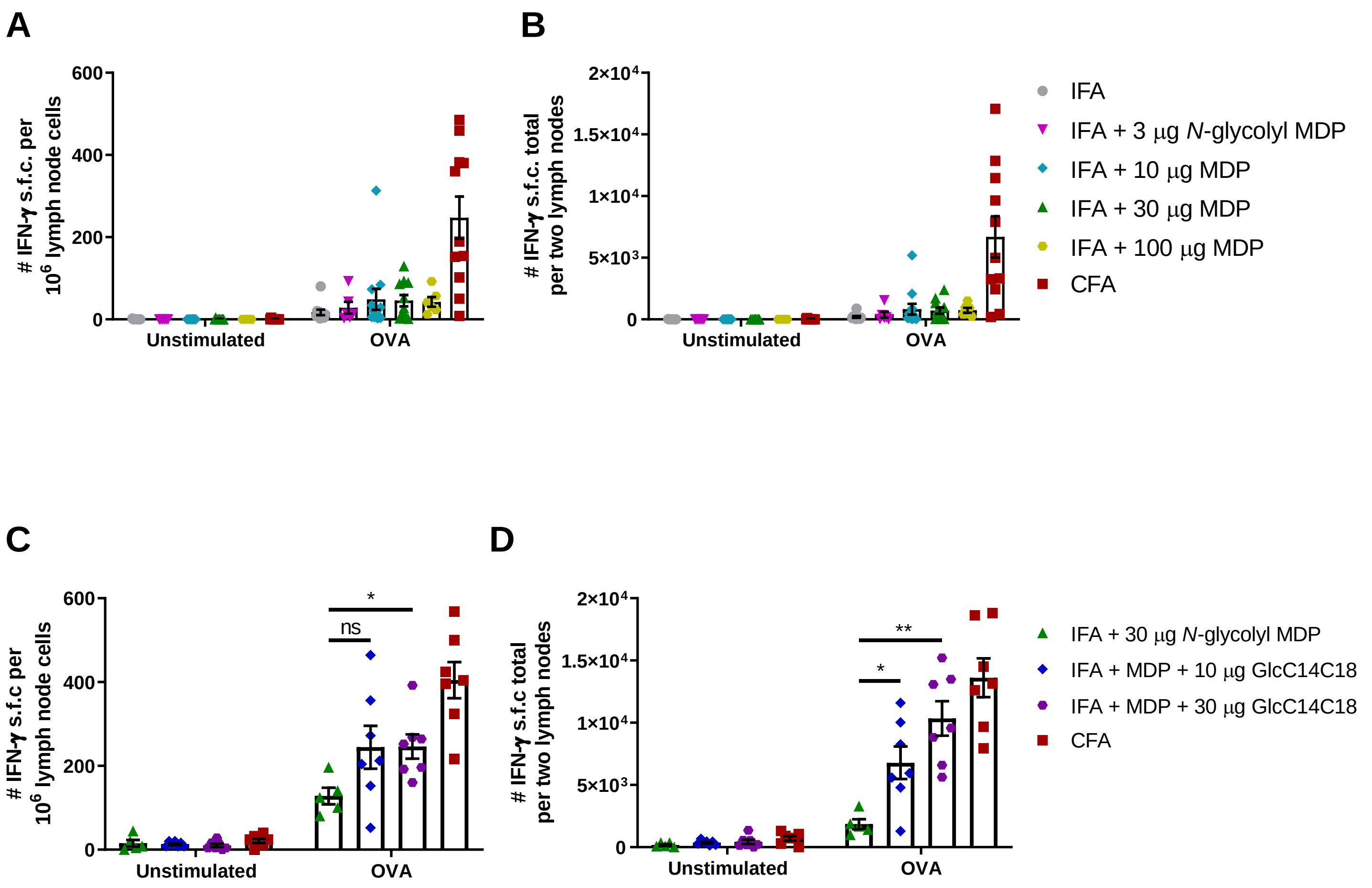

A
600
400
200
0
IFN-γ s.f.c. per 10^6 lymph node cells
Unstimulated
OVA
B
2×10^4
1.5×10^4
1×10^4
5×10^3
0
IFN-γ s.f.c. total per two lymph nodes
Unstimulated
OVA
IFA
IFA + 3 μg N-glycolyl MDP
IFA + 10 μg MDP
IFA + 30 μg MDP
IFA + 100 μg MDP
CFA
C
600
400
200
0
IFN-γ s.f.c per 10^6 lymph node cells
*
ns
Unstimulated
OVA
D
2×10^4
1.5×10^4
1×10^4
5×10^3
0
IFN-γ s.f.c total per two lymph nodes
**
*
Unstimulated
OVA
IFA + 30 μg N-glycolyl MDP
IFA + MDP + 10 μg GlcC14C18
IFA + MDP + 30 μg GlcC14C18
CFA

FIG. S7

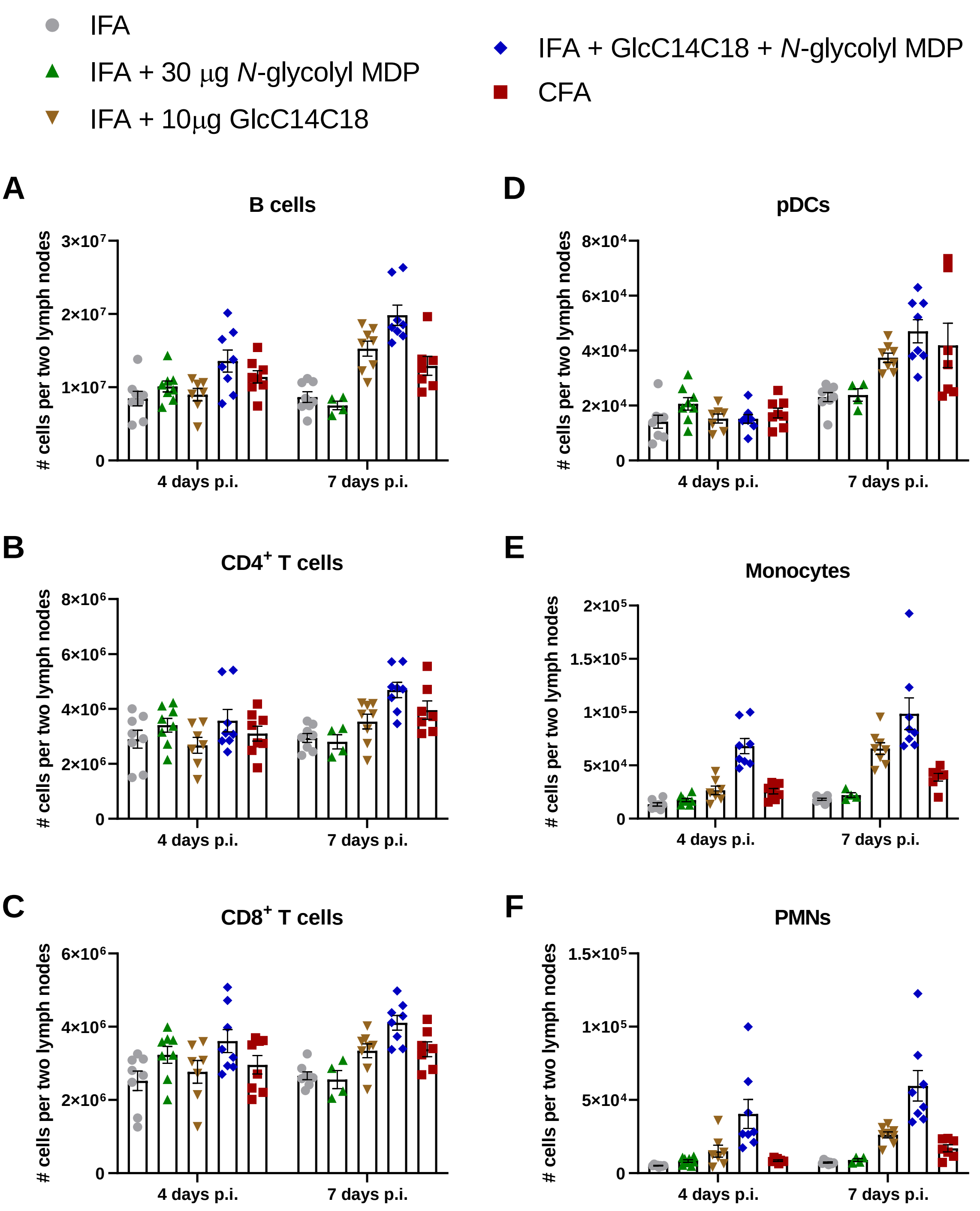

IFA
IFA + 30 µg *N*-glycolyl MDP
IFA + 10µg GlcC14C18
IFA + GlcC14C18 + *N*-glycolyl MDP
CFA
A
B cells
cells per two lymph nodes
3×10⁷
2×10⁷
1×10⁷
0
4 days p.i.
7 days p.i.
D
pDCs
8×10⁴
6×10⁴
4×10⁴
2×10⁴
B
CD4⁺ T cells
8×10⁶
6×10⁶
4×10⁶
2×10⁶
E
Monocytes
2×10⁵
1.5×10⁵
1×10⁵
5×10⁴
C
CD8⁺ T cells
6×10⁶
4×10⁶
2×10⁶
F
PMNs
1.5×10⁵
1×10⁵
5×10⁴

FIG. S8

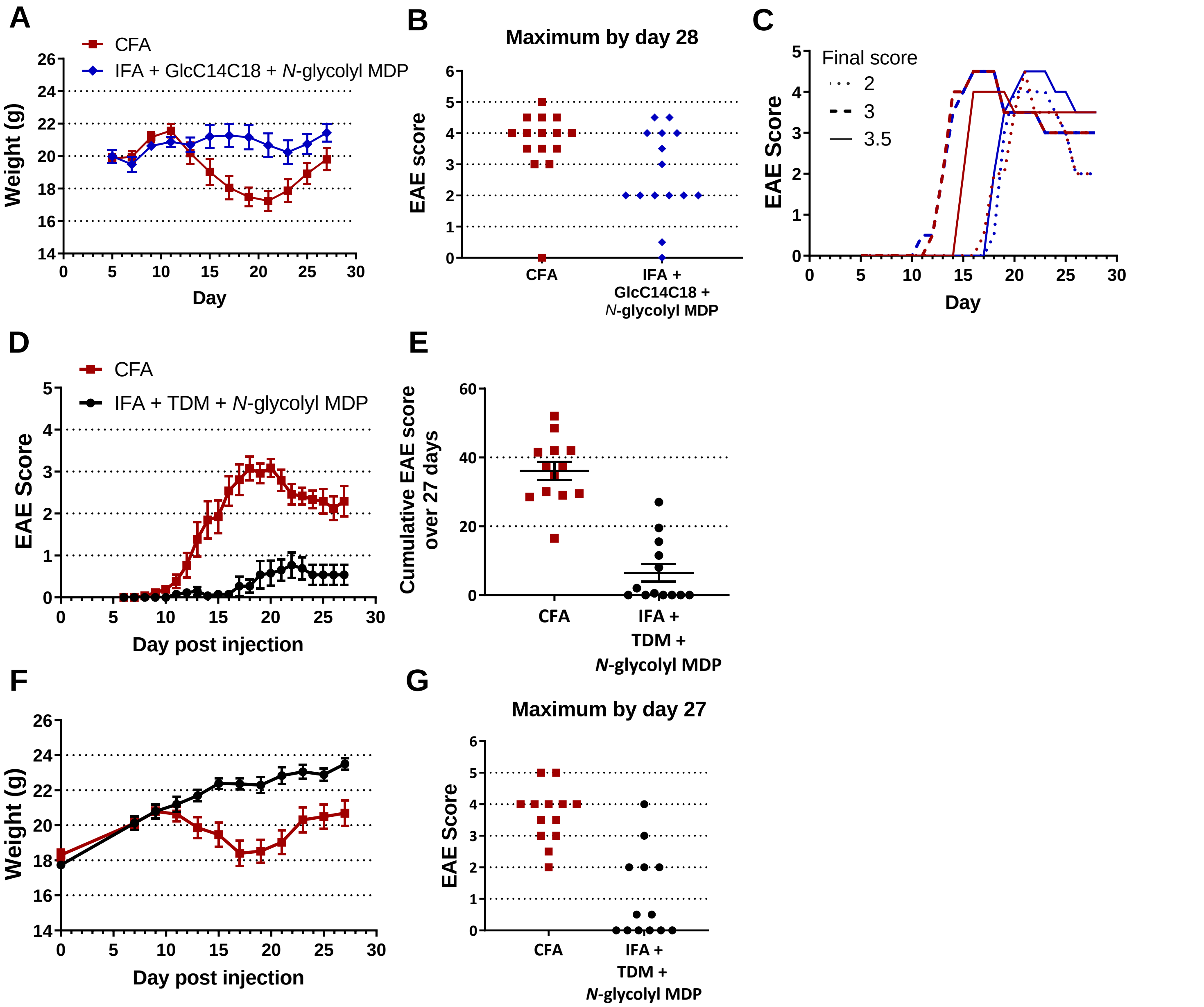

A
CFA
IFA + GlcC14C18 + N-glycolyl MDP
Weight (g)
Day
B
Maximum by day 28
EAE score
CFA
IFA + GlcC14C18 + N-glycolyl MDP
C
Final score
2
3
3.5
EAE Score
Day
D
CFA
IFA + TDM + N-glycolyl MDP
EAE Score
Day post injection
E
Cumulative EAE score over 27 days
CFA
IFA + TDM + N-glycolyl MDP
F
Weight (g)
Day post injection
G
Maximum by day 27
EAE Score
CFA
IFA + TDM + N-glycolyl MDP
